# Factors contributing to the accumulation of reproductive isolation: A mixed model approach

**DOI:** 10.1002/ece3.3093

**Published:** 2017-06-20

**Authors:** Dean M. Castillo

**Affiliations:** ^1^ Department of Biology Indiana University Bloomington IN USA

**Keywords:** comparative methods, reproductive isolation, speciation, sympatry

## Abstract

The analysis of large datasets describing reproductive isolation between species has been extremely influential in the study of speciation. However, the statistical methods currently used for these data limit the ability to make direct inferences about the factors predicting the evolution of reproductive isolation. As a result, our understanding of iconic patterns and rules of speciation rely on indirect analyses that have clear statistical limitations. Phylogenetic mixed models are commonly used in ecology and evolution, but have not been applied to studies of reproductive isolation. Here I describe a flexible framework using phylogenetic mixed models to analyze data collected at different evolutionary scales, to test both categorical and continuous predictor variables, and to test the effect of multiple predictors on rates and patterns of reproductive isolation simultaneously. I demonstrate the utility of this framework by re‐analyzing four classic datasets, from both animals and plants, and evaluating several hypotheses that could not be tested in the original studies: In the *Drosophila* and Bufonidae datasets, I found support for more rapid accumulation of reproductive isolation in sympatric species pairs compared to allopatric species pairs. Using *Silene* and *Nolana*, I found no evidence supporting the hypothesis that floral differentiation elevates postzygotic reproductive isolation. The faster accumulation of postzygotic isolation in sympatry is likely the result of species coexistence determined by the level of postzygotic isolation between species. In addition, floral trait divergence does not appear to translate into pleiotropic effects on postzygotic reproductive isolation. Overall, these methods can allow researchers to test new hypotheses using a single statistical method, while remedying the statistical limitations of several previous methods.

## 1 INTRODUCTION

The divergence of lineages, in the process known as speciation, is only complete after gene flow is reduced following the evolution of reproductive isolation. The two general modes of reproductive isolation are prezygotic and postzygotic barriers to reproduction. Patterns of reproductive isolation inferred from comparative analyses of species willingness or ability to mate (i.e., prezygotic isolation) and produce viable and fertile offspring (i.e., postzygotic isolation) have generated important and influential observations about the evolution of reproductive isolation. These studies have been especially important for making generalizations about comparisons between allopatric and sympatric species pairs, or prezygotic and postzygotic barriers to reproduction (Coyne & Orr, 1989; Funk, Nosil, & Etges, 2006; Malone & Fontenot, 2008; Meiners & Winkelmann, 2012; Mendelson, 2003; Moyle, Olson, & Tiffin, 2004; Presgraves, 2002). These analyses have produced iconic patterns and rules of speciation including that reproductive isolation accumulates more quickly between sympatric species pairs compared to allopatric species pairs and, specifically, that in sympatry prezygotic reproductive isolation appears more quickly than postzygotic reproductive isolation. Nonetheless, the contemporary methods used to analyze these data are known to suffer from both statistical errors and analytical limitations that prevent a more robust simultaneous assessment of the factors that most strongly influence the accumulation of reproductive isolation.

The two methods most typically used to analyze comparative data on reproductive isolation are Mantel tests, including partial mantel tests (Mantel, 1967; Smouse, Long, & Sokal, 1986), and phylogenetic regression based on independent contrasts (PICs; Felsenstein, 1985; Grafen, 1989). These two methods (Mantel and matrix regression vs. PICs) reflect differences in the scale of relationships between lineages that are being tested. Studies that use PICs, or node‐based averages (Fitzpatrick & Turelli, 2006), generally have some information about phylogenetic relationships across the clade or use pairs of sister species, which is needed to accurately calculate independent contrasts. The use of PICs, or methods that assume independence of lineages, is not appropriate for intraspecies data (Felsenstein, 2002; Stone, Nee, & Felsenstein, 2011). Thus, Mantel tests are applied when specific phylogenetic relationships are unknown (if few molecular markers are used) and when the lineages being tested include extensive sampling from only a few closely related species. The main limitations of these approaches are threefold: lack of statistical power, failure to deal adequately with nonindependence, and the inability to test categorical variables or multiple variables simultaneously. The first two limitations have been discussed in detail for the Mantel test, and it has been determined that Mantel tests can have unacceptably high type‐I error rates (Harmon & Glor, 2010; Legendre, 2000). The last limitation results in the inability to test biologically interesting hypotheses, and applies equally to both Mantel test and PIC type tests. For example, in many of the classic studies, the comparisons between sympatric vs. allopatric rates of evolution were never formally tested with a statistical model. Recent attempts to refine the patterns of the accumulation of reproductive isolation have also included other explanatory variables, including ecological differences, range size overlap, and other traits (Turelli, Lipkowitz, & Brandvain, 2014; Yukilevich, 2012), but have not tested multiple variables simultaneously or the interaction between these variables.

Categorical variables are of particular interest to most researchers as they can describe geographic relationships (allopatry vs. sympatry), mating systems (animals: monogamy vs. polygamy, plants: outcrossing vs. selfing), or phenotypic traits (e.g., pigmentation, patterning) that could have very strong influence on the rate and strength of isolation accumulation. Categorical variables can be difficult to model in Mantel tests because they rely on pairwise distance matrices that require no missing data (Mantel, 1967). When categorical variables can be represented as distances, multiple matrix regression can be used for analysis (Legendre & Fortin, 2010; Wang, 2013). This is typically only occurs with binary categorical variables because creating predictor variables based on distances for categorical variables with multiple levels becomes unfeasable. Therefore, Mantel tests based on pairwise distance cannot accommodate hypotheses that test differences between levels of categorical variables.

Regression models are the most promising for incorporating categorical variables, but the analysis is not simple when the regression is carried out using PICs. Continuous variables can be accommodated in PIC models because they are assumed to evolve under Brownian motion; this is what enables node values to be estimated and contrasts to be evaluated. No analogous model exists for categorical variables, unless all daughter taxa (taxa derived from a common ancestor) share the same categorical trait value (Burt, 1989). Even with this conservative approach, the number of contrasts would be reduced tremendously in studies of reproductive isolation and may not be applicable to most systems. One way of circumventing this issue would be to analyze the cross product of a categorical and continuous variable (Garland, Harvey, & Ives, 1992). Although this method would take the model a step further by analyzing differences in slope (assuming genetic distance is a covariate), it cannot be used to estimate mean reproductive isolation because regression in PICs is constrained to pass through the origin.

To accommodate more complex hypotheses, a more appropriate and powerful analytical framework should: (1) have the flexibility to test explicit hypotheses with multiple variables, both continuous and categorical, and (2) able to handle different data types to cover differences in taxonomic scale. Here I describe a framework that overcomes several limitations of current approaches to analyzing these data. Specifically, this framework can be used to test whether geographical context, or any other trait hypothesized to be important to speciation, contributes to patterns of reproductive isolation using comparative crossing data and phylogenetic linear mixed effect models (similar to phylogenetic least squares, PGLS). Using linear models affords the flexibility to test many different hypotheses and can include multiple predictors and their interactions simultaneously. The advantage of this framework over PICs is that the phylogenetic structure is modeled as a covariance matrix, and thus, contrasts for categorical predictors do not have to be calculated. The use of this covariance matrix accounts for phylogeny by either using a pairwise distance matrix or a phylogeny.

I use this framework to reanalyze classic datasets (*Drosophila,* Bufonidae, *Silene*) making explicit statistical comparisons between allopatric and sympatric conditions as an example of how categorical variables can be included in these types of analyses. I test the hypothesis that the rate of accumulation of reproductive isolation differs between these geographic contexts. This test allows new insight into how the process of speciation may be driven by species interactions. In addition, I test two new hypotheses about the relationship between floral differences and reproductive isolation in each of the plant genera *Silene* and *Nolana*. Floral differences are most commonly thought to contribute to prezygotic isolation because flower color and shape (together making up a pollination syndrome; Fenster, Armbruster, Wilson, Dudash, & Thomson, 2004) can determine premating reproductive isolation through pollinator preference (Kay & Sargent, 2009; Schemske & Bradshaw, 1999) and mechanical isolation, including pollen placement (Grant, 1992; Hodges & Arnold, 1994; Smith & Rausher, 2007). New hypothesis have focused on how pollination syndromes can contribute to postmating isolation if there are differences in the ability of pollen to reach the ovary depending on differences in style length (Lee, Page, McClure, & Holtsford, 2008). Moreover, genes that control floral development are active throughout several developmental processes including gametogenesis and embryonic development (Smaczniak, Immink, Angenent, & Kaufmann, 2012), so that postzygotic isolation may evolve as a by‐product of floral divergence (Haak et al., 2014). I use the comparative approach to ask whether divergence in floral morphology significantly increases postmating and postzygotic reproductive isolation.

## 2 METHODS

### 2.1 The need for phylogenetic correction in comparative studies of reproductive isolation

**Figure 1:**
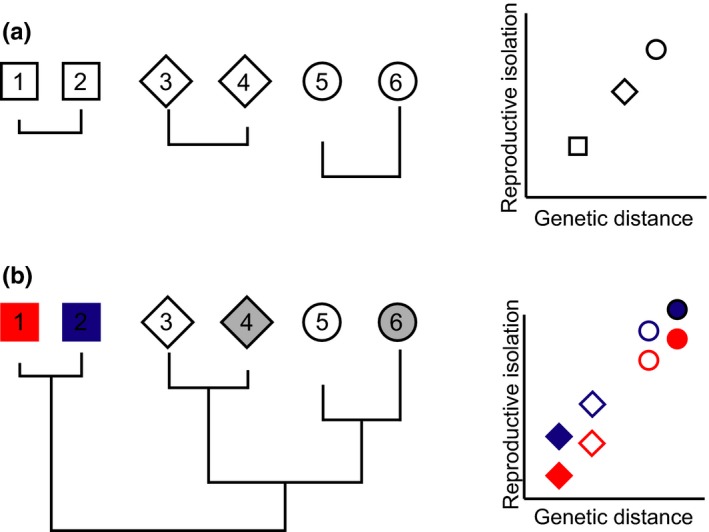
Common experimental designs to evaluate patterns of reproductive isolation and how this trait correlates with divergence time. (a) Species pairs are used, and each pair is considered independent. Each point in the scatterplot represents a single species pair cross. (b) When species are used in multiple crosses, each cross is no longer independent, but the phylogenetic information can be used to account for shared evolutionary history. Each point represents an individual cross. Crosses are color coded by maternal species. The shape and fill of the point represents the paternal species

As species diverge, they are expected to accumulate behaviors and genetic changes that contribute to reproductive isolation. The level of reproductive isolation is predicted to increase as a function of divergence time, with species that are more divergent to have higher levels of reproductive isolation (Figure 1A). Divergence time is often estimated by genetic distance between species. The simplest crossing design to capture this pattern is to use species pairs. In this design, each species is only crossed with one other species generating independent points (Figure 1A). A more realistic design for reproductive isolation experiments involves using each species in multiple different crosses (Figure 1B). In this scenario, phylogenetic correction is necessary because the outcome of crosses involving closely related species are not independent and might reflect shared evolutionary history. For example, when comparing reproductive isolation in a cross between Species 1 and Species 3 with a cross between Species 2 and Species 3, the level of reproductive isolation between these two crosses may be similar because of the shared ancestry of Species 1 and Species 2 (Figure 1B). Including the identity of each parental species used in the cross as a random effect and assuming the variance in the species level random effects are correlated, in a manner predicted by their phylogenetic relationships, in a statistical model accounts for the repeated use of a species in multiple crosses and shared evolutionary history (see below for details).

### 2.2 Description of linear mixed model approach

In their most basic form, linear mixed models include a response variable (*y*), fixed effects (*X*β), random effects (*Zu*), and residuals (ε), where *X* is the fixed effect design matrix, β is the vector of estimated coefficients, *Z* is the random effect design matrix, and *u* is a vector of random effect estimators.

(1) y=Xβ+Zu+ε

Relatedness either via pedigree or phylogeny is incorporated into a structured matrix (typically *A*, the additive genetic relatedness matrix, see (Hadfield, 2010; Hadfield & Nakagawa, 2010) that is considered known and contributes to defining the variance structure of random effects in the model. In equation (2), the random effect estimators are distributed normally with mean = 0 and variance matrix *G*. This matrix can be decomposed into a standard variance–covariance matrix *V*, and the structured matrix *A* (Equation (3))

(2) u∼N(0,G)

(3) G=V×A

This modeling framework can also accommodate a pairwise genetic distance matrix (*D*) to account for nonindependence based on its similarities to the additive genetic relatedness matrix. Each element of *A*, in the absence of inbreeding, represents the expected proportion of genes shared by two individuals. Values range from 0 (completely unrelated individuals) to 1 (completely identical individuals). Whereas some measures of genetic distance are not bound by 0 and 1, (Euclidean distances, Nei's standard genetic distance (Nei, 1972)), others formally meet this requirement (Nei's D_A_ distance (Nei, Tajima, & Tateno, 1983), Wier and Cockerhamθ (Weir & Cockerham, 1984)), and in most actual studies, values are rarely seen above 1. Genetic distance values of 0 represent more closely related populations. To make the genetic distance matrix analogous to the structured *A* matrix I use 1‐*D* in my analyses so that smaller values (closer to 0) represent more distantly related species. Genetic distance used as a predictor variable remained unmanipulated.

The datasets I analyze include *Drosophila* (Coyne & Orr, 1989; Nosil, 2013; Turelli et al., 2014; Yukilevich, 2012), Bufonidae (Malone & Fontenot, 2008), *Silene* (Moyle et al., 2004), and *Nolana* (Jewell, Papineau, Frefre, & Moyle, 2012). All of these datasets have been analyzed previously for patterns of reproductive isolation and are explained in detail below.

### 2.3 Model descriptions

The phylogenetic mixed model framework can be applied to any case where the goal is to test the effect of a categorical variable or multiple variables on the evolution of reproductive isolation. I illustrate this with several examples. In one set of analyses, I estimate the effects of a continuous trait (genetic distance) and a categorical trait (allopatry vs. sympatry; or trait presence vs. absence) and their interaction. In another analysis, I analyze three continuous variables simultaneously (genetic distance, geographic distance, and a phenotypic distance). The random phylogenetic effects of species used in the cross serve two purposes: to account for nonindependence due to phylogeny and account for variance that results from using individual species in multiple crosses in a dataset.

Genetic distance as a predictor variable represents the divergence between the two taxa used in a given cross and should not be confused with the pairwise genetic distance matrix (above) that can account for nonindependence between crosses due to shared evolutionary histories. Even if the exact same numerical values are present in the genetic distance predictor variable and the pairwise genetic distance matrix, these data are used independently in the model and are not redundant. The genetic distance predictor variable is used to model the relationship between reproductive isolation and divergence time (i.e., used to estimate a regression coefficient). The genetic distance pairwise matrix is used as part of the variance–covariance matrix of the random phylogenetic effect for the species of interest.

#### 2.3.1 Model incorporating relatedness and categorical biogeography

A specific model that can be analyzed to determine whether reproductive isolation is different in crosses between sympatric species compared to allopatric species, and how this relationship changes with time since divergence, will incorporate the reproductive isolation data (the measure of either prezygotic isolation, postzygotic isolation, or total isolation) as a function of genetic distance and geographic context (sympatry vs. allopatry) while controlling for phylogenetic relatedness (Equation (4)).

(4) $$y_{\mathrm{RI}}=\mu +x_{1}\beta_{\mathrm{gen}.\mathrm{dist}.}+x_{2}\gamma_{\mathrm{sym}}+\left(x_{1}*x_{2}\right)\beta_{\mathrm{int}}+Z_{f}f+Z_{M}m+e$$

The first term (μ) is the intercept of the linear model and can be thought of as the baseline level of reproductive isolation if there is no significant relationship between reproductive isolation and genetic distance. If there is a significant relationship, the intercept then represents the reproductive isolation between closely related species. The variable *x*_*1*_ is a vector of genetic distance between the species pair, and β_gen.dist._ is the slope of the relationship between reproductive isolation and genetic distance. The variable *x*_*2*_ is a dummy variable (0 when the species pair is allopatric and 1 when the species pair is sympatric), and γ_sym_ is the potential difference in reproductive isolation that can be attributed to the species occurring in sympatry. β_int_ is the potential change in the slope of the relationship between reproductive isolation and genetic distance for sympatric species crosses. Lastly there are two *Z* matrices; they represent the identity of the female parent species and male parent species used in the cross (or can be designated species 1 and species 2 if sex of the parents is not important). I use a separate effect for each parent in the cross because not all species are used as both a male parent and female parent, depending on the dataset. Using separate matrices reduces the sparseness of each matrix and improves the precision of the estimates for the variance of the random effects. Recent studies use interactions between phylogenetic effects for species interactions (Hadfield, Krasnov, Poulin, & Nakagawa, 2014), but for the datasets in this study, there are few crosses made outside of very closely related species so any matrix describing an interaction between phylogenetic effects would be very sparse and hard to estimate. Using this model, I was able to test for differences in the rate of reproductive isolation for three datasets: *Drosophila,* Bufonidae, and *Silene* (described below). The *Drosophila* dataset has previously been shown to have increased levels of prezygotic reproductive isolation between sympatric pairs compared to allopatric pairs (Coyne & Orr, 1989), but these rates were never directly compared in a single model.

#### 2.3.2 Model incorporating relatedness and categorical trait differences

As a second example, I incorporate a categorical variable with more than two levels, such as floral color differences or similarities between the species pair, using the same model described above but with flower color substituted for geographic context.

(5) $$y_{\mathrm{RI}}=\mu +x_{1}\beta_{\mathrm{gen}.\mathrm{dist}.}+x_{2}\gamma_{\mathrm{color}}+\left(x_{1}*x_{2}\right)\beta_{\mathrm{int}}+Z_{f}f+Z_{m}m+e$$

Here, the *x*_*2*_ variable can have two levels, for example, *x*_*2*_ will have two levels if one level represents crosses between species that share the same state for floral color (red × red and white × white) and the other level represents crosses between species with different floral colors (red × white and the reciprocal). Alternatively the *x*_*2*_ variable could have multiple levels: crosses between species that both have red flowers, crosses between species that both have white flowers, and crosses between red and white flowered species. This model would test whether specific floral colors or morphologies have increased speciation rates compared to other floral morphologies.

#### 2.3.3 Model incorporating multiple continuous variables with potential correlations

Often geographical context may not be captured by a categorical variable (allopatry vs. sympatry) but instead by a continuous variable such as geographic distance. When spatial variation is included in analyses, we expect correlations between geographic variables and other variables of interest including genetic distance. We expect that lineages that are more geographically isolated are also more genetically differentiated. Additional correlations may exist between morphological traits that can contribute to reproductive isolation, such as quantitative floral traits. This model allows multiple continuous variables to be analyzed simultaneously and accounts for correlations between the predictor variables (Equation (5)). In the example illustrated below, I include genetic distance, geographic distance, and the difference in corolla tube length, which is one measure that captures the difference in floral size between species.

(6) $$y_{\mathrm{RI}}=\mu +x_{1}\beta_{\mathrm{gen}.\mathrm{dist}.}+x_{2}\beta_{\mathrm{geo}.\mathrm{dist}}+x_{3}\beta_{\mathrm{corolla}}+Z_{f}f+Z_{m}m+e$$

If these variables are correlated with one another, it will be difficult to make inferences on any given parameter estimate as multicollinearity can cause variance inflation (increased estimates of variance parameters compared to model where variables are not correlated). To address this issue, I explicitly allow for covariance between these variables in the model by changing the prior structure of the predictor variables (fixed effects) from being independent (Equation (7)), to being correlated. This is reflected in the joint prior distribution being a covariance matrix, where nondiagonal elements represent covariance between predictor variables (Equation (8)).

(7) $$\left(\begin{array}{c} \beta_{1}\\ \beta_{2}\\ \beta_{3} \end{array}\right)∼\left(\begin{array}{ccc} \sigma_{1}^{2} & 0 & 0\\ 0 & \sigma_{2}^{2} & 0\\ 0 & 0 & \sigma_{3}^{2} \end{array}\right)$$

(8) $$\left(\begin{array}{c} \beta_{1}\\ \beta_{2}\\ \beta_{3} \end{array}\right)∼\left(\begin{array}{ccc} \sigma_{1}^{2} & \sigma_{1,2}^{2} & \sigma_{1,3}^{2}\\ \sigma_{2,1}^{2} & \sigma_{2}^{2} & \sigma_{2,3}^{2}\\ \sigma_{3,1}^{2} & \sigma_{3,2}^{2} & \sigma_{3}^{2} \end{array}\right)$$

This model can be applied to the *Nolana* dataset, for example, to disentangle the effects of multiple correlated variables. In the original analysis of the *Nolana* dataset (Jewell et al., 2012), there was some evidence that genetic distance a geographic distance predicted reproductive isolation. However, as these two variables were highly correlated that analysis lacked power to disentangle this pattern using partial mantel tests. Using the approach here, I directly modeled the correlation between the continuous variables by including a covariance matrix between the three continuous variables.

#### 2.3.4 Model incorporating genetic distance matrix in place of phylogeny

Another way to correct for relatedness in this framework is to use a pairwise genetic distance matrix. This method would be most suitable for closely related species for which constructing a phylogenetic tree is inappropriate. As an example, I analyzed the *Nolana* data using information from a pairwise genetic distance matric (specifically 1‐*D*, where *D* is the genetic distance matrix) in the place of the structured *A* matrix, to see whether the inferences varied between the two methods. The package MCMCglmm (Hadfield, 2010) requires the inverse of the structured matrix and has built in functions to take the inverse of the relatedness matrix from either pedigree or phylogeny information. To use the genetic distance matrix, I used the ginv function from the MASS (Venables & Ripley, 2002) package to find the generalized inverse of the 1‐*D* matrix.

### 2.4 Interpreting model outputs

In the Bayesian framework used in MCMCglmm, there is no formal distinction between fixed and random effects, but for ease of explanation, I will call all parameters that are estimated for the predictor variables fixed effects, and the phylogenetic variance random effects. I can test any specific hypothesis for fixed effects by examining the highest posterior density (HPD) of the parameter from a given model. For example, to test the hypothesis that there is a difference in average reproductive isolation between allopatric and sympatric species pairs, I determine whether the HPD for regression coefficient ($\gamma_{\mathrm{sym}}$) includes 0. If $\gamma_{\mathrm{sym}}$ does not include 0, then there is evidence supporting the hypothesis of a significant difference in reproductive isolation between allopatric and sympatric species pairs. Similarly, testing the hypothesis that the rate of accumulation of reproductive isolation differs between two groups (e.g., sympatric and allopatric pairs) involves evaluating the slope $\beta_{\mathrm{int}}$ to again assess whether the HPD includes 0. For all analyses, I set the prior distributions for random effect covariances and residuals as follows. MCMCglmm uses inverse Wishart distributions for random effects, with scale parameter *V*, and degrees of freedom parameter *n*. For each model, I set *V* to be 1/3 the total variance in the response variable because there were three covariance matrices (two matrices for male and female parent, and the residual covariance matrix).

For each model, I ran two MCMC chains; this enabled me to determine lack of convergence by examining within and between chain properties. The trace plots (iteration number vs. value of the draw) were visually inspected for all variables and chains, to see whether chains were mixing well or whether there was high auto‐correlation (which would signify chain being stuck on a local maximum and thereby give a false signal of convergence). Along with visual inspection, the primary tool used to determine whether the MCMC chains failed to converge was the Gelman–Rubin Diagnostic (Gelman & Rubin, 1992). This test uses information from the variance of the mixture of both chains, and the variance within a single chain to calculate a potential reduction factor (Brooks & Gelman, 1998; Gelman & Rubin, 1992). A value of 1 indicates that the chains have converged because the ratio of variance between and within chains is identical. In practice, chains are typically run until the reduction factor for all variables is <1.1 (Brooks & Gelman, 1998). I used the coda package (Plummer, Best, Cowles, & Vines, 2006) in R to calculate the convergence diagnostics. I considered a model to have converged if the all scale reduction factors for all variables (both fixed and random effects) were ≤1.1 and the trace plots indicate good mixing (actual scale reduction factors were generally <1.06, and the multivariate scale reduction factors were <1.02.) Code for all analyses is available through Dryad digital repository.

### 2.5 Datasets

Four different datasets were used here and are explained in detail below; as outlined, in several cases, these were enriched with additional tree construction, biogeographical information, and trait data prior to performing analyses. All datasets and phylogenies are available Dryad digital repository.

#### 2.5.1 Drosophila

**Figure 2:**
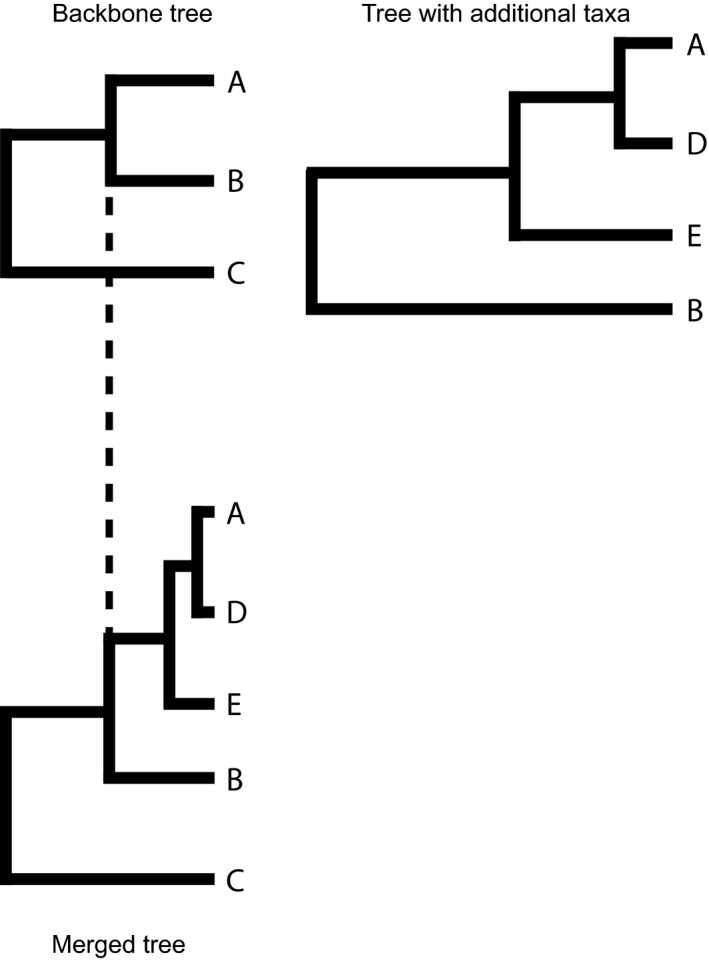
A visual representation of how more detailed information about a species group found in one tree can be integrated into a larger phylogeny by transforming branch lengths. In this example, the relationships between species A, D, E, and B are scaled to the same length that occurred in the backbone tree (for the clade consisting of species A and B)

The *Drosophila* dataset is an expansion of the original dataset used by Coyne and Orr (Coyne & Orr, 1989) and was accessed from http://www.drosophila-speciation-patterns.com/. In this dataset, prezygotic isolation estimates are based on choice and no‐choice mating assays, depending on the specific species pair. Postzygotic isolation is a combination of the hybrid sterility or hybrid inviability of F1 progeny produced from the cross. To include phylogenetic information in my model, I combined data from two phylogenies that had complementary information and largely agreed on phylogenetic relationships. The first phylogeny (van der Linde, Houle, Spicer, & Steppan, 2010; VL) provides a useful backbone for different species groups, but lacks species richness within some groups. The second phylogeny (Morales‐Hojas & Vieira, 2012; MH) includes more species for particular clades. As the VL tree includes more species groups in total, I used this tree to define relationships between the major species groups. The VL tree was constructed from a supermatrix, and I was able to combine it with the MH as follows. After scaling both trees so that they were ultrametric, I could substitute species relationships from the MH tree into the VL tree, by transforming the branch length from the MH tree, so they were proportional to the branch length of the corresponding clade in VL tree (see, e.g., Figure 2). For some groups, reproductive isolation data included races or subspecies and these were represented as polytomies. After constructing the phylogeny, I only retained crossing data where both species were present in the phylogeny, leaving me with 182 crosses total to include in analyses.

#### 2.5.2 Bufonidae

The primary dataset consisted of postzygotic isolation estimated from in vitro crosses (Malone & Fontenot, 2008). Several stages of development were used to calculate a reproductive isolation index including: fertilization rate, hatching rate, number of tadpoles produced, percentage of tadpoles metamorphosed, fertility in backcross analysis, and the stage at which eggs ceased to develop. The reproductive isolation index was then calculated similarly to Coyne and Orr (1989) and Presgraves (2002). To evaluate the sensitivity of the inferences to this index, I also conducted analyses on an additional reproductive isolation index that takes into account that these barriers are sequential (Ramsey, Bradshaw, & Schemske, 2003). This made the response variable more continuous instead of considering only a fixed number of values.

I used the original sequence alignment to construct a neighbor‐joining tree replicating the original analysis (Malone & Fontenot, 2008). In addition, I also enriched this dataset by determining allopatry and sympathy relationships, by downloading shape files (vectors storing geometric information) for each species from the IUCN Red List Database (IUCN 2014.). These files were reprojected (changed from 3D to 2D objects) to Albers Equal areas using rgdal (Bivand, Keitt, & Rowlingson, 2013) and maptools (Bivand & Lewin‐Koh, 2013) in R with parameters specific for the region where they were located (Asia, Africa, Europe, or North America). I then determined whether species ranges overlapped using PBSmapping (Schnute, Boers, & Haigh, 2012) in R. Species that had no overlap were designated as allopatric.

#### 2.5.3 Silene

The original crossing data were compiled in Moyle et al. (2004). Prezygotic isolation was a measure of the total number of failed pollinations (likely due to pollen pistil interactions) in interspecific crosses, compared to the reproductive isolation of the parental species. Postzygotic isolation was estimated from pollen sterility of F1 hybrids. The original data included sympatric and allopatric relationships and I enriched the dataset by including flower color for each species. Floral color data were summarized from the available literature, online flora projects, and personal observations. The phylogeny comes from a supertree (Jenkins & Keller, 2011). Similarly to the *Drosophila* data, I only retained taxa for analysis that could be placed into the phylogeny and I allowed polytomies for certain taxa where different subspecies were used in crosses. This yielded 65 crosses for analyses.

#### 2.5.4 Nolana

The *Nolana* data were originally presented in Jewell et al. (2012). As the authors found no measurable prezygotic isolation, I focused on total postzygotic isolation that was a combination of fruit set, mericarp size, and seed set. The phylogeny used in the original study had several large polytomies. To resolve these polytomies, I used the original sequence data to construct a new phylogeny in Raxml (Stamatakis, 2014), by allowing each gene to have its own substitution model. Genes on the chloroplast were concatenated and treated as one unit. These data also contained a complete pairwise genetic distance matrix, pairwise measures of geographic distance, and pairwise measures of differences in specific aspects of floral morphology, all three of which are significantly correlated with one another. In my analyses, I used corolla diameter differences to quantify floral distance, although similar results were achieved with another measure of floral divergence (corolla depth difference), likely because these measures were highly similar and correlated (data not shown).

## 3 RESULTS

### 3.1 Drosophila

Using the *Drosophila* reproductive isolation data, I tested the hypothesis that there are differences in the level of reproductive isolation between allopatric and sympatric species pairs (μ = baseline for allopatry and $\gamma_{\mathrm{sym}}$ = change in RI for sympatry) and that reproductive isolation accumulates at a different rate between allopatric and sympatric species pairs (i.e., slope between genetic distance and RI differs; $\beta_{\mathrm{gen}.\mathrm{dist}.}$ = relationship between genetic distance and RI for allopatric pairs and $\beta_{\mathrm{int}.}$ = change in slope for sympatric pairs). To interpret these models, it is often easiest look at which coefficients contribute to reproductive isolation in allopatric vs. sympatric species pairs. The overall model has many coefficients but only a few differentiate the allopatric vs. sympatric species pairs. The overall model has the following form:

(9) $$y_{\mathrm{RI}}\;=\;\mu \;+\;x_{1}\beta_{\mathrm{gen}.\mathrm{dist}.}+\;x_{2}\gamma_{\mathrm{sym}}\;+\;\left(x_{1}*x_{2}\right)\beta_{\mathrm{int}}\;+\;Z_{f}f\;+\;Z_{m}m\;+\;e$$

The variable *x*_*1*_ is a continuous variable, but the variable *x*_*2*_ is binary and is equal to 0 for allopatric pairs and 1 for sympatric pairs. Thus, for allopatric pairs, the model simplifies to:

(10) $$y_{\mathrm{RI}}\;=\;\mu \;+\;x_{1}\beta_{\mathrm{gen}.\mathrm{dist}.}\;+\;Z_{f}f\;+\;Z_{m}m\;+\;e$$

For sympatric pairs, the model simplifies to

(11) $$y_{\mathrm{RI}}\;=\;\mu \;+\;\gamma_{\mathrm{sym}}\;+\;x_{1}\left(\beta_{\mathrm{gen}.\mathrm{dist}.}+\;\beta_{\mathrm{int}}\right)\;+\;Z_{f}f\;+\;Z_{m}m\;+\;e$$

This highlights that the coefficient β_int_. represents the change in slope for sympatric pairs and that the total slope for sympatric species pairs is the sum of the two beta coefficients.

**Figure 3:**
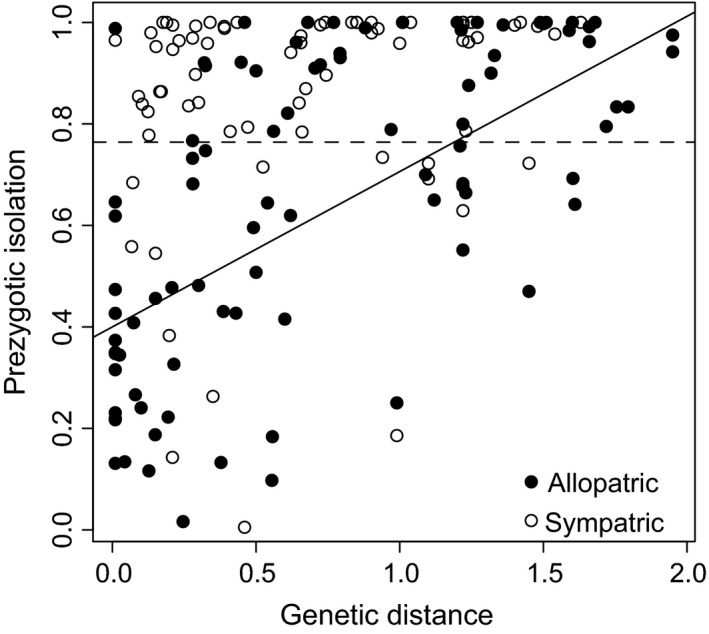
The relationship between genetic distance and prezygotic isolation for *Drosophila* species pairs that are either allopatric or sympatric, demonstrating that most sympatric species pairs show almost complete isolation, resulting in no relationship between genetic distance and reproductive isolation for the sympatric context. The best fit lines were constructed using the mode of the parameters from the MCMCglmm analysis. The solid line represents allopatric species pairs, and the dashed line represents sympatric species pairs

For prezygotic isolation, the intercept (baseline level of reproductive isolation) was significantly different than zero, and reproductive isolation was significantly elevated in sympatric pairs ($\gamma_{\mathrm{sym}}$ = (0.2533, 0.4764), (lower 95% HPD interval, upper 95% HPD interval); Table 1). There was also a significant relationship between genetic distance and reproductive isolation, as detected in the original study (Coyne & Orr, 1989), but only for allopatric species pairs. In comparison, the overall relationship between genetic distance and reproductive isolation is nonsignificant for sympatric pairs (Table 1). In this model, the coefficients are additive (Equation (11)), and the relationship between genetic distance and reproductive isolation is not significantly different from zero (HPD for $\beta_{\mathrm{gen}.\mathrm{dist}}+\beta_{\mathrm{int}}$ = (−0.1250, 0.3571)). The effect of sympatry on reproductive isolation ($\gamma_{\mathrm{sym}}$) is so strong that most of the reproductive isolation values are near 1 across all distances (completely isolated; Figure 3).

**Table 1:** Summary of coefficients estimated for the analysis of prezygotic (left) and postzygotic (right) reproductive isolation from the *Drosophila* data. The confidence intervals are for 95% highest posterior density (HPD) and are significant if they do not include zero (in bold)

Coefficient	Biological meaning	Prezygotic		Postzygotic	
		Lower	Upper	Lower	Upper
μ (intercept)	Average RI	**0.1975**	**0.6033**	−0.0005	0.4001
β_gen.dist._	Slope relating genetic distance and RI	**0.1959**	**0.4164**	**0.1906**	**0.4644**
$\gamma_{\mathrm{sym}}$	Additional RI in sympatry	**0.2533**	**0.4764**	−0.1162	0.1355
$\beta_{\mathrm{int}}$	Increase in slope for sympatry	−**0.3209**	−**0.0593**	**0.1326**	**0.5247**

For postzygotic isolation, the intercept was not significantly different than zero; neither was the increase in reproductive isolation in sympatry were not significantly different than zero (μ and $\gamma_{\mathrm{sym}}$ had HPD overlapping zero; Table 1). This indicates that there is little to no postzygotic isolation in recently diverged species regardless of geographical context. The relationship between genetic distance and reproductive isolation ($\beta_{\mathrm{gen}.\mathrm{dist}}$) was significant, and the rate of increase of reproductive isolation with genetic distance was greater in sympatric pairs ($\beta_{\mathrm{int}}$ = 0.1326, 0.5247). This suggests that reproductive isolation may accumulate more quickly between sympatric pairs of species than allopatric pairs, and the difference increases as divergence time (genetic distance) increases.

### 3.2 Bufonidae

Analyses of the Bufonidae data using the two alternative indices of reproductive isolation were qualitatively the same (Table 2), so I only discuss results using the original index of reproductive isolation. The model intercept was significantly different than zero (μ = 0.2980, 0.6945), and the effect of sympatry was to actually decrease the level of reproductive isolation ($\gamma_{\mathrm{sym}}$ = −0.2816, −0.0427) although the overall level of reproductive isolation was still nonzero (the HPD for μ + $\gamma_{\mathrm{sym}}$ = (0.0164,0.6518)). The relationship between genetic distance and reproductive isolation was quite steep ($\beta_{\mathrm{gen}.\mathrm{dist}}$ = 3.6212, 5.8819), and the increased rate of accumulation in sympatric pairs was also significant ($\beta_{\mathrm{int}}$ = 0.4679, 3.5163). In combination, these coefficients suggest that even though there is little reproductive isolation for very recently diverged sympatric pairs (those separated by small genetic distances), reproductive isolation accumulates more quickly for sympatric pairs than allopatric pairs.

**Table 2:** Summary of coefficients estimated for the analysis of postzygotic reproductive isolation from the Bufonidae data. The original index of reproductive isolation (left) was calculated by Malone and Fontenot (2008) following the procedure of Coyne and Orr (1989) and Presgraves (2002). The new index (right) takes into account that reproductive barriers are sequential following Ramsey et al. (2003). The confidence intervals are for 95% highest posterior density (HPD) and are significant if they do not include zero (in bold)

Coefficient	Biological meaning	Postzygotic (Original)		Postzygotic (New)	
		Lower	Upper	Lower	Upper
μ (intercept)	Average RI	**0.2980**	**0.6945**	**0.8398**	**0.9686**
β_gen.dist._	Slope relating genetic distance and RI	**3.6212**	**5.8819**	**0.4472**	**1.2592**
$\gamma_{\mathrm{sym}}$	Additional RI in sympatry	−**0.2816**	−**0.0427**	−**0.0988**	−**0.0105**
$\beta_{\mathrm{int}}$	Increase in slope for sympatry	**0.4679**	**3.5163**	**0.2665**	**1.3992**

### 3.3 Silene

Sympatry had no effect on the baseline prezygotic reproductive isolation ($\gamma_{\mathrm{sym}}$ = −0.3643, 0.1510) or on the rate of accumulation of reproductive isolation ($\beta_{\mathrm{int}}$ = −1.5671, 3.5080), which is consistent with the original results from Moyle et al. (2004). There was a significant relationship between genetic distance and reproductive isolation ($\beta_{\mathrm{gen}.\mathrm{dist}}$), which did not differ between sympatry and allopatry (Table 3). The lack of allopatric pairs for the postzygotic and total reproductive isolation measurements precluded analysis of the effects of geographical context on these measures, although the general relationship between reproductive isolation and genetic distance was positive and significant consistent with Moyle et al. (2004); data not shown.

**Table 3:** Summary of coefficients estimated for the analysis of prezygotic reproductive isolation when considering geographical context (left) or floral divergence (right) from the *Silene* data. The confidence intervals are for 95% highest posterior density (HPD) and are significant if they do not include zero (in bold)

Coefficient	Biological meaning	Prezygotic (Geographic model)	
		Lower	Upper
μ (intercept)	Average RI	−1.2138	1.9094
β_gen.dist._	Slope relating genetic distance and RI	**0.9126**	**7.1054**
$\gamma_{\mathrm{sym}}$	Additional RI in sympatry	−0.3643	0.1510
$\beta_{\mathrm{int}}$	Increase in slope for sympatry	−1.5671	3.5080

Coefficient	Biological meaning	Prezygotic (Floral differences model)	
		Lower	Upper
μ (intercept)	Average RI	−1.0166	1.4672
β_gen.dist._	Slope relating genetic distance and RI	**3.4368**	**9.5154**
$\gamma_{\mathrm{color}}$	Additional RI due to color differences	−0.1556	0.3158
$\beta_{\mathrm{int}}$	Increase in slope for color differences	−2.4138	2.2990

**Figure 4:**
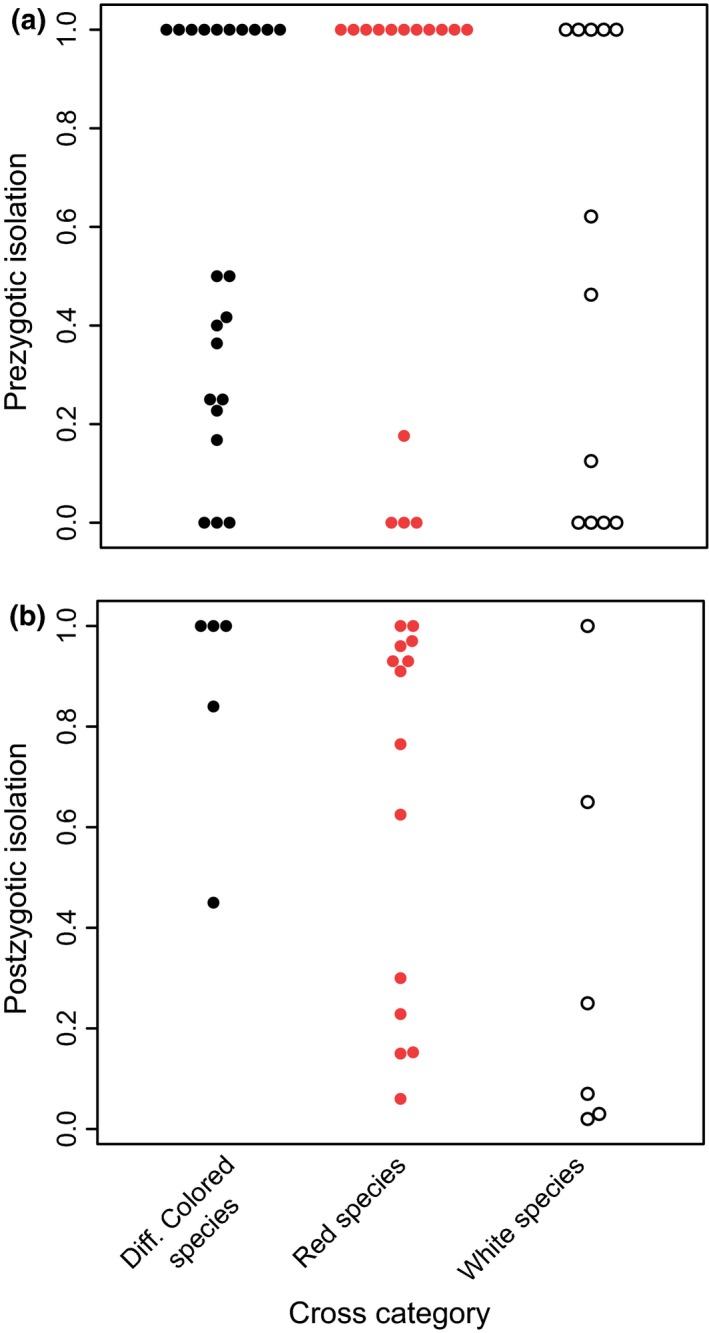
Variation in the level of reproductive isolation between *Silene* species pairs that either had different floral colors or shared floral colors (separated into pairs where both species were either white or red) for both (a) prezygotic and (b) postzygotic isolation demonstrating no effect of floral differences on reproductive isolation

Floral color differences did not increase reproductive isolation, no matter which index of reproductive isolation you consider. Regardless of the cross category (crosses between species that differed in floral color vs. between species that had the same color), there was a large amount of variation in reproductive isolation with some pairs having little to no isolation, and other pairs having substantial isolation (Figure 4). Thus, there was no effect of floral color on the average levels of reproductive isolation or on the accumulation of reproductive isolation over time, and the only significant effect was genetic distance on reproductive isolation (Table 3; prezygotic model shown).

### 3.4 Nolana

**Figure 5:**
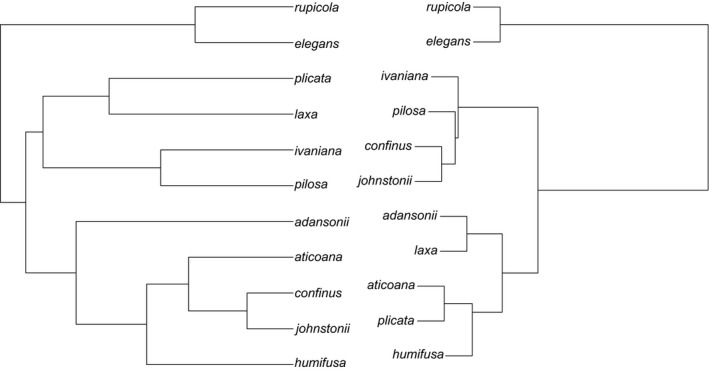
Comparison of relationships in the genus *Nolana* when either a phylogeny made from several loci or matrix of pairwise genetic distances is used. The topology on the left represents the maximum‐likelihood tree based on sequence from the *ADH2, atpB, ndhF, psbA‐trnH, rps16* genes. The topology on the right was generated using Ward's hierarchical clustering method for pairwise genetic distances reported in Jewell et al. (2012)

When genetic distance, geographic distance, and measures of flower size differences were considered jointly, the only significant predictor of reproductive isolation was geographic distance ($\beta_{\mathrm{geo}.\mathrm{dist}}$ = 0.0001, 0.0004; note, the small coefficient is due to the scale of reproductive isolation/kilometers), signifying that there is more reproductive isolation between pairs of species that are more geographically distant. This is consistent with the original study in the sense that only geographical distance was significant using the Mantel test (Jewell et al., 2012). The analysis using the genetic distance matrix also indicated that geographic distance was the only significant predictor of reproductive isolation (Table 4), and the coefficients were similar to the previous analysis. The main difference was there was no significant intercept (μ = (−1.0166, 1.4672)). This is likely caused by the differences in inferred relatedness between the phylogeny and the genetic distance matrix (Figure 5), as this was the only difference in the two models. In a phylogeny relatedness is based on shared ancestry, whereas a distance matrix includes all nucleotide changes without context of whether they are shared with other taxa or phylogenetically informative. The result of using the genetic distance matrix to infer relatedness may have been to infer that more closely related species had little reproductive isolation, so the intercept was not significantly different than zero.

**Table 4:** Summary of coefficients estimated for the analysis of total reproductive isolation when using the phylogeny (left) or genetic distance matrix (right) from the *Nolana* data. The confidence intervals are for 95% highest posterior density (HPD) and are significant if they do not include zero (in bold)

Coefficient	Biological meaning	Total reproductive isolation (phylogenetic matrix)		Total reproductive isolation (genetic distance matrix)	
		Lower	Upper	Lower	Upper
μ (intercept)	Average RI	**0.1166**	**0.6249**	−0.3126	0.4921
$\beta_{\mathrm{gen}.\mathrm{dist}.}$	Slope relating genetic distance to RI	−0.3149	0.4861	−0.4088	0.3945
$\beta_{\mathrm{geo}.\mathrm{dist}.}$	Slope relating geographic distance (km) to RI	**0.0001**	**0.0004**	**0.0001**	**0.0004**
$\beta_{\mathrm{corolla}}$	Slope relating differences in corolla diameter to RI	−0.0071	0.0140	−0.0106	0.0115

## 4 DISCUSSION

Understanding how reproductive isolation accumulates over time in different geographic contexts (allopatry vs. sympatry) or according to specific trait differences requires analyzing the interaction between genetic distance and these factors and their joint effects on reproductive isolation. These comparisons cannot be made using PICs or Mantel tests, so in many classical studies, the effects of these factors are instead indirectly compared (Coyne & Orr, 1989, 1997; Jewell et al., 2012; Moyle et al., 2004). Using a phylogenetic mixed model framework, and classic datasets on species ability to cross with one another, I formally tested the interactions among these effects on the evolution of reproductive isolation. I explicitly examined interactions between categorical and continuous variables and accounted for correlations between predictor variables, something that is not possible in the standard PICs framework (Burt, 1989; Garland et al., 1992) and difficult to implement using Mantel tests when there are more than two levels of the categorical variable. Mantel tests only evaluate the correlation between two variables but not average effects (i.e., intercepts), which must be tested with other methods, whereas estimates of mean differences between two groups are simultaneously estimated using a mixed model approach.

In addition, in the mixed modeling framework, the correlation between predictor variables can be modeled and accounted for through specific variance matrices, which enables several predictors to be tested simultaneously where multicollinearity may have previously limited inferences (as demonstrated in the analysis of *Nolana*). In comparison, current approaches to evaluating the effect of multiple variables on reproductive isolation have clear limitations. Partial Mantel tests do not examine multiple effects simultaneously, but instead evaluate what additional variance a variable may explain after accounting for variance due to other variables (Smouse et al., 1986). Although multiple variables can be accommodated in a standard regression using PICs, it is be difficult to determine the significance of coefficients when the analysis involves correlated independent variables because multicollinearity causes spurious variance inflation (Mundry, 2014). This is especially troubling, given that the nature of the data used in these studies will often contain correlations between the variables that may explain the accumulation of reproductive isolation.

The phylogenetic mixed effect model is also flexible in that relationships between taxa can be conveyed either through a phylogenetic relatedness matrix or a genetic distance matrix. In contrast, often the use of PICs vs. Mantel test reflects the scale of relatedness that researchers are examining: PICs typically examine interspecific data using phylogenies while Mantel tests can focus on intraspecific data using genetic distance matrices. The mixed model approach is a natural way to apply these analyses across different phylogenetic scales.

### 4.1 New insights from phylogenetic mixed model framework

For the *Drosophila* and Bufonidae analyses, I specifically tested whether geographic context (allopatry or sympatry) influences the rate of accumulation of reproductive isolation. These data were both originally analyzed using linear regression (similar to PICS); however, the difference in the rate of accumulation (represented by the slope relating genetic distance to reproductive isolation) could not be tested directly because of the inability to calculate contrasts for binary variables, and therefore, the interaction between geographic context and genetic distance could not be evaluated. Using the phylogenetic mixed model enabled direct tests of the differences in the rate of isolation accumulation, and doing so produced new insights into these previously analyzed datasets. In particular, I found that in both *Drosophila* and Bufonidae, there was an increase in the rate of accumulation of postzygotic reproductive isolation in sympatric species pairs compared to allopatric species pairs. This result contrasts with the analysis by Coyne and Orr (1997) and likely reflects the fact that Coyne and Orr (1997) used a subset of the data that only included recently diverged species (D < 0.25), whereas analyses here included the entire dataset. Other studies that have included data from both sympatric and allopatric pairs in Lepidoptera (Presgraves, 2002) and birds (Price & Bouvier, 2002) failed to find differences between sympatry and allopatry in the level of reproductive isolation, although the inferences were based on informal analyses.

For prezygotic isolation, the presence of stronger prezygotic isolation among closely related species pairs in sympatry is often assumed to be a product of reinforcement (Yukilevich, 2012), but reinforcement unlikely to contribute directly to increased accumulation of postzygotic isolation in sympatry (Servedio, 2000; Servedio & Sætre, 2003). An alternative hypothesis, called the Templeton Effect or differential fusion effect, proposes that strong reproductive isolation in sympatry is a consequence of a systematic bias among species pairs that are able to maintain their integrity in sympatry, whereby weakly isolated species fail to maintain species boundaries (and therefore undergo species collapse), leaving only species pairs with strong reproductive isolation. This hypothesis has previously been proposed to explain patterns of strong prezygotic isolation in sympatry (Noor, 1999; Templeton, 1981; Yukilevich, 2012); however (unlike reinforcement), the effect may be equally applicable to postzygotic isolation. The templeton‐differential fusion effect may be reinterpreted and applied to postzygotic isolation such that the level of postzygotic isolation that exists between species will determine the likelihood of species coexistence. This is because sexual isolation alone is often not strong enough to maintain species barriers (Lande, 1981; Payne & Krakauer, 1997; Servedio & Burger, 2014), and reinforcement is sensitive to gene flow. If the strength of postzygotic isolation between sympatric lineages drives reinforcement and ultimately the strength of prezygotic isolation (Servedio, 2000; Servedio & Sætre, 2003), we should observe stronger postzygotic isolation in sympatry (as observed for both the *Drosophila* and Bufonidae datasets) and a correlation between postzygotic and prezygotic isolation in sympatry. Interestingly, postzygotic and prezygotic isolation are correlated in sympatry in the *Drosophila* dataset (Yukilevich, 2012).

The mixed model approach can also be used to evaluate the effect of categorical trait variation on the strength and accumulation of reproductive isolation, to address additional mechanistic questions. In my analyses of whether floral divergence can contribute to postmating and postzygotic reproductive isolation, independent of effects on pollinator visitation, I found no support in *Silene* for the hypothesis that floral divergence could also influence these reproductive isolation phenotypes through pleiotropic effects (Haak et al., 2014). Specifically, floral divergence in the form of flower color differences did not contribute to either prezygotic isolation (most likely via pollen–pistil interactions) or postzygotic isolation (F1 pollen sterility). It is possible that the pleiotropic effects of floral traits on reproductive isolation could be manifested in a different trait (seed sterility, F1 germination, or F1 viability) or floral divergence could contribute to extrinsic postzygotic isolation (Lowry, Modliszewski, Wright, Wu, & Willis, 2008; Ramsey et al., 2003), none of which were captured in this analysis. Alternatively, differentiation in floral traits other than color might be more important in this context. In *Silene*, for example, there is some evidence that floral traits other than red vs. white flower color, including flower display height and orientation (Brothers & Atwell, 2014; Fenster, Reynolds, Williams, Makowsky, & Dudash, 2015) and floral scent (Castillo, Kula, Dötterl, Dudash, & Fenster, 2014; Waelti, Muhlemann, Widmer, & Schiestl, 2008), may contribute to reproductive isolation via pollinator visitation.

Like floral variation, the variation in other traits might also be correlated with other factors that contribute to reproductive isolation. For example, if floral shape has phylogenetic signal, then floral divergence would be correlated with genetic distance; similarly, if there is selection for different flower sizes in different environments, floral divergence would be associated with geographical distance. Under these scenarios, floral divergence might seem to be contributing to reproductive isolation, merely because it is correlated with a factor that drives the accumulation of reproductive isolation. It is important to be able to distinguish these potential mechanisms. In the original *Nolana* analysis (Jewell et al., 2012), floral, geographical, and genetic distance measures were all observed to be correlated. In the reanalysis here, I did not observe an independent effect of floral divergence (floral size difference) on reproductive isolation. Indeed, I was able to explicitly rule out the possibility that floral changes contributed to reproductive isolation while simultaneously testing the effects of genetic distance and geographic distance and found geographical distance alone contributed to reproductive isolation in this system. From this analysis, it is clear that differences in flower size do not appear to translate into pleiotropic effects on reproductive isolation in this system, as has been hypothesized for differences in traits associated with pollinator preference (Haak et al.^2014^).

## 5 CONCLUSION

The phylogenetic mixed model framework utilized in this study remedies difficulties for Mantel tests and PICs in testing hypothesis about factors contributing to the evolution of reproductive isolation. To demonstrate the utility of this framework, I performed several analyses to evaluate the roles of categorical geographic and trait variation, and quantitative divergence measures, on the accumulation and strength of isolation in four published datasets. I tested the role of geography in the evolution of reproductive isolation and was able to show that reproductive isolation accumulates more quickly in sympatry not only for prezygotic isolation but also for postzygotic isolation. In the datasets examined, floral traits did not contribute to the pattern or strength of reproductive isolation measures included in the original studies. This framework can enable future studies to test complex hypothesis, test the effects of multiple variables simultaneously (even if they are correlated), and use a generalized framework to examine reproductive isolation between species or at the intraspecies level.

## DATA ACCESSIBILITY

Data Archival location: Dryad Digital Repository doi:10.5061/dryad.gf83v

## CONFLICT OF INTEREST

None declared.
